# A host-microbial metabolite interaction gut-on-a-chip model of the adult human intestine demonstrates beneficial effects upon inulin treatment of gut microbiome

**DOI:** 10.20517/mrr.2023.79

**Published:** 2024-02-22

**Authors:** Joanne M. Donkers, Maria Wiese, Tim J. van den Broek, Esmée Wierenga, Valeria Agamennone, Frank Schuren, Evita van de Steeg

**Affiliations:** ^1^Department of Metabolic Health Research, TNO, Leiden 2333 BE, the Netherlands.; ^2^Department of Microbiology & Systems Biology, TNO, Leiden 2333 BE, the Netherlands.

**Keywords:** Gut-on-a-chip, microbiome, *in vitro* models, *ex vivo* tissue, host-response, microbial metabolite, short-chain fatty acids, host-microbe interaction

## Abstract

**Background:** The gut and its microbiome have a major impact on many aspects of health and are therefore also an attractive target for drug- or food-based therapies. Here, we report on the added value of combining a microbiome screening model, the i-screen, with fresh intestinal tissue explants in a microfluidic gut-on-a-chip model, the Intestinal Explant Barrier Chip (IEBC).

**Methods:** Adult human gut microbiome (fecal pool of 6 healthy donors) was cultured anaerobically in the i-screen platform for 24 h, without and with exposure to 4 mg/mL inulin. The i-screen cell-free culture supernatant was subsequently applied to the luminal side of adult human colon tissue explants (*n* = 3 donors), fixed in the IEBC, for 24 h and effects were evaluated.

**Results:** The supplementation of the media with inulin promoted the growth of *Anaerostipes*, *Bifidobacterium*, *Blautia*, and *Collinsella* in the *in vitro* i-screen, and triggered an elevated production of butyrate by the microbiota. Human colon tissue exposed to inulin-treated i-screen cell-free culture supernatant or control i-screen cell-free culture supernatant with added short-chain fatty acids (SCFAs) showed improved tissue barrier integrity measured by a 28.2%-34.2% reduction in FITC-dextran 4000 (FD4) leakage and 1.3 times lower transport of antipyrine. Furthermore, the release of pro-inflammatory cytokines IL-1β, IL-6, IL-8, and TNF-α was reduced under these circumstances. Gene expression profiles confirmed these findings, but showed more profound effects for inulin-treated supernatant compared to SCFA-supplemented supernatant.

**Conclusion:** The combination of i-screen and IEBC facilitates the study of complex intestinal processes such as host-microbial metabolite interaction and gut health.

## INTRODUCTION

Gut health, or intestinal health, has a major impact on our overall health and well-being. Not only is the gastrointestinal system the main portal for nutrients and thus for energy and building blocks for our body, but it also acts as a barrier of defense against disease and harmful substances and is very communicative with our brain and other organs via its own nervous system^[1-3]^. Furthermore, the gut hosts the largest microbial community in the human body, with the highest microbial cell density of around 10^11^ CFUs/mL being present in the proximal colon^[4,5]^. In return for nutrients and intestinal mucus provided by the host, the microbiota produces compounds beneficial for the intestinal cells and body, such as vitamins, neurotransmitters, and short-chain fatty acids (SCFAs)^[6-8]^. By doing so, and via other mechanisms, it supports the protection against pathogens, enhances the immune system, influences gut-brain communication, and impacts the gut epithelial cells^[6,8,9]^. The eubiosis of the gut microbial community and a proper balance between the microbiota and the host are very important for good health. In fact, several pathological diseases such as autoimmune disorders, allergies, and IBD have been associated with a disbalance in the host-microbe homeostasis^[10,11]^. The six main gut microbial phyla are *Bacillota*, *Bacteroidota*, *Actonomycetota*, *Pseudomonadota*, *Fusobacteriota*, and *Verrucomicrobiota*, of which the first two represent approximately 90% of the total gut microbiota^[7,12]^. The majority of the gut microbiota are strict anaerobes, which dominate the facultative anaerobes and aerobes by two to three folds^[4]^. The microbiome plays an important role in food fermentation, and in the proximal colon, the microbiome mainly converts non-digestible carbohydrates and dietary fibers into SCFA^[13]^. The main SCFAs produced by the microbiota are acetate, propionate, and butyrate^[14]^. Over the recent years, SCFAs have gained increasing attention for local beneficial effects in the gut such as improved gut barrier function and reduced intestinal inflammation^[9,14-17]^. Higher intestinal levels of SCFAs may be achieved by increased consumption of dietary fiber; however, the efficiency of fiber fermentation and the subsequent production of metabolites depends on the composition and functional capabilities of the gut microbiota and the specific dietary fiber or composition of non-digestible carbohydrates that are being provided^[18]^. Such substrates, selectively utilized by microorganisms and leading to host health benefits, are also called prebiotics^[16,19]^. Inulin is a well-known example of a prebiotic; its strong bifidogenic and SCFA-inducing effects have been described by us previously^[20]^ and many others^[21-24]^. Therefore, manipulation of the gut microbiome is likely to be a physiologically adequate strategy to increase SCFA production in the gut^[16,25]^. *In vitro* or *ex vivo* models are very effective to test such hypotheses^[26,27]^. Here, we connect the microbial component to host gut tissue in a host-microbial metabolite interaction gut-on-a-chip model of the adult human large intestine. Fresh human adult colon tissue explants were exposed in our microfluidic *ex vivo* tissue model, the Intestinal Explant Barrier Chip (IEBC)^[28]^, to cell-free culture supernatant from our *in vitro* intestinal microbiota screen, the i-screen^[29]^, without and with inulin intervention to stimulate microbial SCFA production. This sequential use of an *in vitro* model and an *ex vivo* model provides a unique strategy to study the interaction of microbial metabolites, influenced by specific interventions, with the host tissue in a controlled experimental setting.

## MATERIALS AND METHODS

### I-screen supernatant

#### Fecal collection

Fecal material was provided by six healthy adult volunteers (Caucasian, age 25-65 years, no antibiotic use in the 3 months preceding the donation nor consumed prebiotics or probiotics the week before donation, self-assessment of health status). Collection of fecal samples was performed anonymously following TNO standard operational procedures. The collection was approved by an internal ethical evaluation board and is in compliance with the Dutch laws on medical/scientific research. Participants gave written informed consent. Fecal samples were collected and prepared as described^[30,31]^ with some modifications. In brief, fecal samples were collected by the volunteers with the FecesCatcher (fecesvanger.nl). Fecal material was transferred into a container in an anaerobic jar equipped with an AnaeroGen sachet (Thermo Fisher Diagnostics GMBH). The jar was kept cool and delivered to the laboratory within 24 h. The material was introduced in an anaerobic chamber, diluted 1:3 with phosphate-buffered saline, and homogenized using a Tissue Homogenizer Omni THQ (12-500, Omni International). Finally, 20% glycerol was added before storing the material at -80 °C.

#### Anaerobic incubation: i-screen

The fecal material was incubated in the i-screen (intestinal screening), an *in vitro* system for the anaerobic incubation of fecal microbiota^[29]^. Before starting the i-screen incubations, the six fecal samples were pooled and pre-cultured overnight in a modified standard ileal efflux medium (SIEM) under anaerobic conditions, at 37 °C and with shaking at 300 rpm^[30]^. The microbiota was then transferred to a microtiter plate for incubation in SIEM medium, with and without 4 mg/mL inulin, with pH adjusted to 5.8. The incubation started with a fecal bacterial load of approximately 10^9^ CFU/mL. Conditions were tested in triplicate (pre-culture at t = 0 h), or with *n* = 9 or *n* = 6 replicates for control or inulin-treated conditions, respectively. At the start of the incubation (t = 0, referred to in this article as pre-culture), and after 24 h of fermentation in anaerobic conditions, 100 µL and 50 µL of sample material were collected and used for DNA isolation and metabolite analyses, respectively.

#### DNA isolation

Following incubation, samples were collected and DNA was isolated as described^[32]^.

#### 16S rRNA amplicon sequencing

Changes in the microbiota composition were analyzed by using 16S rRNA gene amplicon sequencing as described^[32]^.

#### Metabolite analyses

SCFAs acetate, propionate, and butyrate, and branched chain fatty acids (BCFA) iso-butyrate and iso-valerate were analyzed as described^[32]^.

#### Data analysis microbiome

Statistical analysis of the microbiome data was performed using R version 4.1.2^[33]^. Figures were composed using the ggplot2 package^[34]^. The phyloseq package was used to manage the phylogenetic sequencing data^[35]^.

Before ordination, the 16S data was filtered to include only those ASVs that contribute to the first 97.5% of all counts in the data. The method for selection of ASVs in this way is described in detail in^[32]^.

Principal component analysis on microbiome data was performed using the pcaMethods package^[36]^. For this analysis, the data were centered log-ratio transformed to account for their compositional properties. To calculate the weighted averages of the SCFAs in the inulin and untreated control conditions of the PCA ordination of the microbiome data, the wascores function from the vegan package was used^[37]^.

A linear mixed-effects model was employed to identify differences in microbial abundance, which facilitated the control for both fixed effects of the experimental conditions and random effects due to sample replication. For this analysis, we used edgeR^[38]^ alongside variancePartition^[39]^ for data normalization and variance modeling, respectively. This modeling approach accounts for between-replicate variability.

Visualization of the differential abundance data was conducted using a heatmap, which displayed log2 fold changes of microbial genera across the experimental conditions.

To determine whether incubation with inulin resulted in significant changes in SCFA production compared to the control condition, a linear model was fitted to data from each of the SCFAs using the lm function. ANOVA testing was performed on these models to make comparisons between the treated samples and the untreated control for each SCFA.

### Intestinal explant barrier chip

#### Chemicals and assay buffer

[^14^C]antipyrine was purchased from American Radiolabeled Chemicals Inc. and [^3^H]atenolol was purchased from Moravek Biochemical Inc., all other chemicals were purchased from Sigma-Aldrich Chemie B.V. unless stated otherwise. Williams E buffer was prepared and used according to Stevens *et al*.^[40]^. Williams E supplemented with 1% penicillin/streptomycin was used for transport and handling of the tissue. Williams E supplemented with 1% and 4% BSA was used to precoat the system and in the basolateral compartment during an experiment, respectively.

#### Processing of i-screen culture supernatant for exposure in the IEBC model

The culture supernatant of inulin-treated and untreated conditions was processed to obtain cell-free culture supernatant containing the microbial metabolites. Therefore, the i-screen culture supernatant was pooled and centrifuged at 3,000 rpm for 15 min, subsequently filtered using a 0.22 µM filter, and stored at -20 °C until use. For the IEBC experiment, this cell-free culture supernatant, from now on just called supernatant, was set at pH 6.5 (based on^[41]^) and supplemented with 25 mM d-glucose, 10 mL/L Glutamax and 10 mL/L HEPES, 50 μg/mL gentamicin, and 25 μg/mL amphotericin. Additionally, for the short-chain fatty acid treatment, the choice of butyrate, acetate, and propionate concentrations was based on the i-screen SCFA metabolite analysis and the SCFAs were added to the supplemented untreated control supernatant in a concentration of 20, 50, and 10 µM, respectively.

#### Human intestinal tissue collection and preparation

Human intestinal proximal colon tissue was obtained from three human adult patients undergoing surgery for colon carcinoma. Informed consent was requested from the patients and ethical approval for the use of human intestinal tissue was provided by the hospital board. Collection and preparation of the tissue explants were described previously^[28]^.

#### Intestinal colon tissue in the intestinal explant barrier chip

The design and fabrication of the IEBC is described by Eslami Amirabadi *et al.*^[28]^. Experiment preparation and execution were as described previously^[28,42]^ with two modifications: no dose-replacement at t = 20 h, and supernatant from i-screen supplemented with FD4, [^14^C]antipyrine, and [^3^H]atenolol was used as an apical medium during the experiment.

#### Assessment of tissue viability

To assess the viability of the *ex vivo* intestinal segments, the cytosolic enzyme lactate dehydrogenase (LDH) was measured in the apical and basolateral supernatants of the two-compartmental model, and homogenized tissue segments, using an LDH kit (Sigma-Aldrich) as described previously^[28,40,43]^. The acceptance criterion for this parameter is leakage <  3% per hour of total LDH under control conditions.

#### Assessment of tissue integrity

Tissue barrier integrity was determined using FITC Dextran 4000 (FD4) as described previously^[28]^. The acceptance criterion for this parameter is FD4 leakage < 0.5% per hour under control conditions.

#### Assessment of tissue functionality/permeability

Tissue functionality was calculated as described^[42]^, here using [^3^H]atenolol (low permeability) and [^14^C]antipyrine (high permeability) as reference markers for the paracellular and transcellular transport route, respectively. The transcellular over paracellular apparent permeability (P_app_) ratio was calculated as P_app_ antipyrine/P_app_ atenolol.

#### Determination of cytokines

After 24 h of incubation, IL-6, IL-8, IL-1β, IL-10, IL-12p70, IL-13, IL-2, IL-4, IFN-γ, TNF-α cytokine release into the apical and basolateral compartments by the intestinal tissue in the IEBC was determined by applying V-PLEX Proinflammatory panel 1 (K15049D) according to the manufacturer’s instructions. Cytokine concentration levels were determined using a Meso Scale Discovery (MSD) Sector Imager 2400 instrument equipped with discovery workbench software (version 3).

#### RNA isolation and RT-qPCR

Total RNA was isolated from approximately 50 mg of human colon tissue with RNAqueous™ Total RNA Isolation Kit (Invitrogen). RNA integrity was assessed spectrophotometrically at 260 nm using a Platereader Synergy H1 (Biotek). Five hundred nanograms of total RNA was used to synthesize first-strand cDNA with iScript™ Reverse Transcription Supermix for RT-qPCR (Bio-Rad). RT-qPCR was carried out in a Quantstudio 6 flex (Applied Biosystems) using iQ" SYBR Green Supermix (Bio-Rad) and was analyzed using Quantstudio Real-Time PCR software. Expression levels in each sample were normalized for the expression level of housekeeping gene 36B4. Relative expression of genes of interest was calculated using the ΔΔCt method. Primer sequences are noted in Table 1.

**Table 1:** Primer sequences

**Gene**	**Forward primer**	**Reverse primer**
*36B4*	TCATCAACGGTACAAACGA	GCCTTGACCTTTTCAGCAAG
*ZO-1*	GCACAGCAATGGAGGAAACAG	CCAAATCCAGGAGCCCTGT
*CLDN-1*	CTTGGAAGACGATGAGGTGCA	CCAGACCTGCAAGAAGAAATATCG
*CLDN-2*	CTCCTGGGATTCATTCCTGTT	TCAGGCACCAGTGGTGAGTAGA
*OCLN*	GCTACGGAAGTGGCTATGG	GCGGCAATGAAACAAAAG
*IL-8*	AGTTTTTGAAGAGGGCTGAGA	TGCTTGAAGTTTCACTGGCATC
*TNSF10*	CGTCAGCTCGTTAGAAAGATGATT	TGGTCCCAGTTATGTGAGCTG
*CCL20*	CAAGAGTTTGCTCCTGGCTG	CAAAGTTGCTTGCTGCTTCT
*HDAC3*	AGTTCTGCTCGCGTTACACA	CAGAAGCCAGAGGCCTCAAA
*LBP*	CAAGGGCATCAGCATTTCGG	TTCAACAGCCACCCCAAGTC
*MUC2*	TaqMan primer probes; Assay ID Hs03005103_g1	
*MUC5B*	TaqMan primers probes; Assay ID Hs00861595_m1	

#### Data analysis IEBC data

Statistical analysis of the microbiome data was performed using R version 4.1.2^[33]^. Figures were composed using the ggplot2 package^[34]^.

Statistical analysis of tissue integrity, functionality & viability data, cytokines, and gene expression was performed using the lme4 and lmerTest packages, with the emmeans package for post hoc analysis^[34,39,44]^. Estimated marginal means were transformed back to their original scale in the case of models with log-transformed variables.

Three separate IEBC experiments were performed, each with multiple replicates. In the case of antipyrine and atenolol, data were collected at multiple time points during a given experiment. Each model has its own random effects structure so that the effects of the experimental condition could be estimated independently of the random variation introduced by the different experimental occasions replicates and sampling time points.

In the case of LDH, we used a hierarchical random effects structure where replicate is uniquely nested within each experiment. For FD4, atenolol, antipyrine, and the atenolol/antipyrine ratio, the time factor was accounted for with an additional separate random effects term. Cytokines and gene expression were measured in a single experiment; the random effects structure was adjusted accordingly with only the replicate factor as a random effect.

The FD4 model used a Gamma distribution with a square-root link function to accommodate the distribution of the underlying data. All models except for FD4 data used log-transformed values to ensure heteroscedastic residuals. Samples were excluded from statistical analysis when their absolute standardized residuals exceeded 3 standard deviations.

## RESULTS

### Impact of inulin on microbial diversity and short-chain fatty acid production

**Figure 1:**
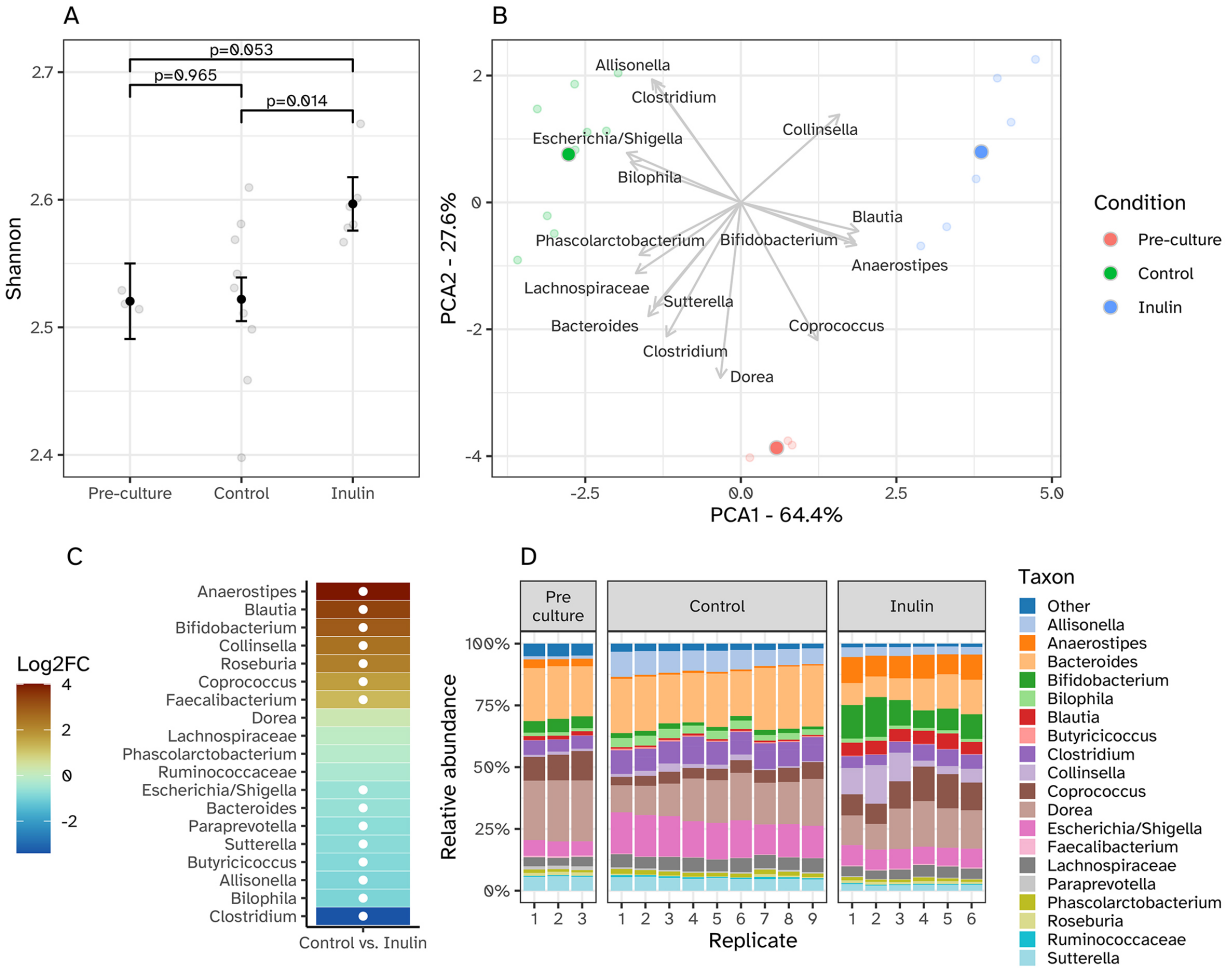
Microbiome data of the i-screen pre-culture (t = 0 h, *n* = 3), the i-screen control, and inulin-supplemented fermentations with pooled microbiota samples (both t = 24 h, *n* = 9 or *n* = 6, respectively). (A) Shannon index showing a significant increase in microbial alpha diversity after incubation with inulin. Black error bars with points represent estimated marginal means with standard errors, obtained from linear mixed-effects models; (B) PCA plot showing the relationship between the microbiota samples and the most abundant bacterial genera identified. The darker dots represent the average per sample type (pre-culture t = 0 h, control t = 24 h, and inulin supplementation t = 24 h), and the lighter dots represent replicates. The position of the dots in the PCA plot is indicative of the microbiota composition of the corresponding samples. Hence, dots that are closer to each other represent samples that are more similar to each other in microbiota composition. The clustering of dots based on color indicates that the microbiota composition of the control samples is distinct from that of the inulin-supplemented samples and that both are different from the pre-culture at t = 0 h; (C) Differential abundance of microbial genera between control and inulin-supplemented fermentations. The heatmap illustrates the log2 fold change (Log2FC) in the abundance of microbial genera between inulin treated and untreated conditions: after 24 h of fermentation in the i-screen. Each row represents a genus, reordered based on the magnitude of Log2FC. Color intensity indicates the degree of increase in relative abundance (yellow) or decrease (blue) relative to the control condition. White circles highlight genera with statistically significant changes (*P* ≤ 0.01). The analysis accounts for variability within replicate samples using a mixed model framework; (D) Relative abundance (%) of the 20 most abundant taxa on genus level for all experimental replicates per condition (pre-culture t = 0 h, control i-screen t = 24 h, inulin supplemented i-screen t = 24 h).

The effect of the inulin intervention on the gut microbial community structure and function *in vitro* was studied after 24 h of incubation. We investigated the inulin-induced change in alpha and beta diversity in the microbial community composition [Figure 1]. A significant increase in the Shannon diversity index was detected after 24 h of fermentation in the i-screen when SIEM media was supplemented with 4 mg/mL inulin (*P* = 0.014) [Figure 1A]. Supplementation of SIEM media with 4 mg/mL of inulin led to an increase in Alpha diversity to 2.60 in Shannon index compared to 2.52 in the control condition which resembled the diversity in the pre-culture at t = 0. Furthermore, the inulin treatment led to an increase in the relative abundance of the genera *Anaerostipes*, *Bifidobacterium*, *Blautia*, *and Collinsella*, as detected and displayed in the PCA plot [Figure 1B and C]. Inulin supplementation also promoted the growth of *Coprococcus* to a larger extent than the control fermentation, while suppressing the relative abundance of *Escherichia* and *Shigella*, *Allisonella*, *Bacteroides*, *Bilophila*, *Clostridium*; these genera were higher in relative abundance in the control condition than in the inulin and pre-culture condition [Figure 1D].

**Figure 2:**
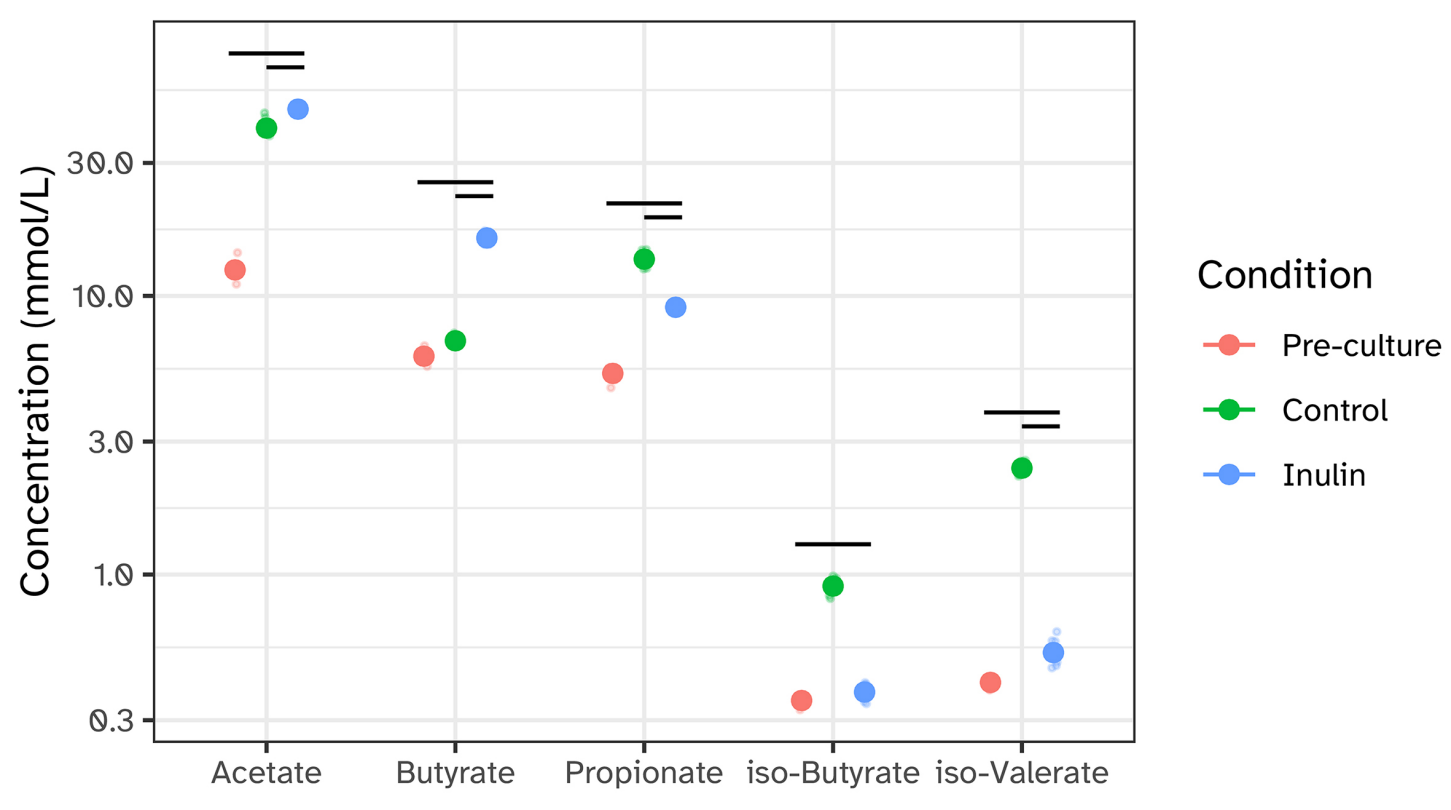
SCFA and BCFA concentration in i-screen. SCFA and BCFA were measured in absolute amounts (mmol/L) in the pre-culture at t = 0 h, or after 24 h of fermentation in the i-screen. The black bars represent statistical comparisons between the different conditions. They indicate where a significant (*P* < 0.05) difference in short-chain fatty acid concentrations was detected. SCFA: Short-chain fatty acid; BCFA: branch-chain fatty acid.

The supplementation of SIEM media with inulin led to a significantly elevated level of butyrate production by the microbiota, with 16.14 ± 0.83 mmol being detected in the supernatant of the inulin-supplemented conditions compared to 6.96 ± 0.22 mmol detected in the control supernatant (*P* < 0.001) [Figure 2]. Acetate levels were only slightly higher in the inulin-supplemented condition with 46.52 ± 0.98 mmol compared to the control with 41.02 ± 2.78 mmol, and propionate levels were slightly lower in the inulin-treated condition with 8.98 ± 0.35 *vs.* 13.51 ± 0.94 mmol in the control condition. In addition to these individual differences, total SCFA levels were significantly higher upon inulin supplementation with 72.56 ± 1.38 mmol *vs.* 63.87 ± 3.32 mmol in the control condition. The relative contributions of each individual SCFA were also significantly different between these two groups, with the most dominant shifts being a 2.1-fold increase for butyrate and a 1.7-fold reduction for propionate.

### Effects of increased butyrate concentrations on the integrity, functionality and viability of human colon tissue

**Figure 3:**
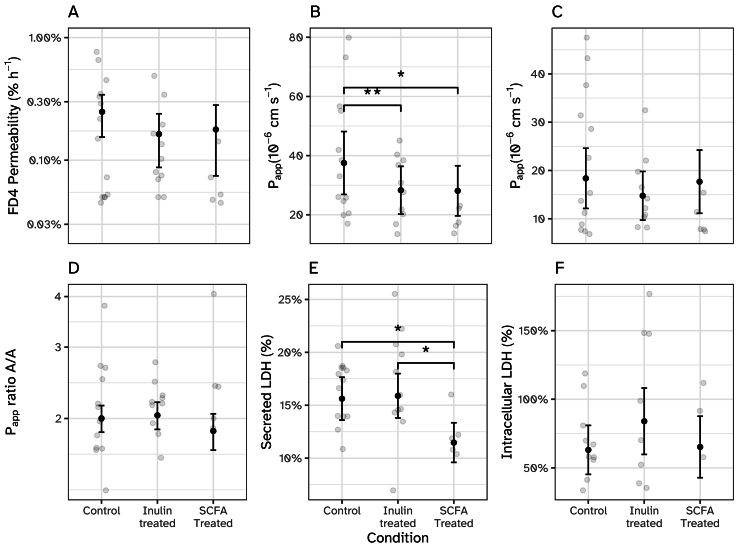
Tissue integrity, functionality and viability of the human colon tissue explants in the IEBC exposed to supernatant from i-screen (untreated control, inulin-treated, and untreated control with added SCFA) for 24 h (*n* = 5-13/group). For i-screen ctrl and i-screen inulin-treated, data were collected from 3 independent experiments; for i-screen ctrl with added SCFA data, were collected from one experiment (donor 3). (A) Average FD4 permeability, expressed as leakage (%)/h, between 20-24 h; (B and C) The average P_app_ of antipyrine (10 μM). (B) and atenolol (10 μM), (C) was calculated between 20-24 h; (D) Ratio of transcellular transport (P_app_ antipyrine) to paracellular transport (P_app_ atenolol); (E) Cumulative LDH release into the apical and basolateral compartments and (F) intracellular LDH were determined after 24 h and compared to the level of intracellular LDH at t = 0 h. Black error bars with points represent estimated marginal means with standard errors, obtained from linear mixed-effects models. *P < 0.05; **P < 0.01. IEBC: Intestinal explant barrier chip; SCFA: short-chain fatty acid; FD4: FITC Dextran 4000; P_app_: apparent permeability; LDH: lactate dehydrogenase.

We investigated the potential beneficial effects of the changed SCFA composition upon inulin treatment of the microbiome, with a shift towards increased butyrate concentrations, by exposing fresh human colon tissue explants, obtained from three different donors, to i-screen cell-free culture supernatant collected from untreated control microbiome or microbiome incubated with inulin. Additionally, in one experiment, a third condition was included, which mimicked the SCFA composition of the inulin-stimulated microbiota by adding a mix of butyrate (20 mM), acetate (50 mM), and propionate (10 mM) to untreated control supernatant. Effects on the tissue explants were evaluated between 20-24 h of incubation [Figure 3]. The ameliorating effect of supernatant containing higher levels of butyrate on epithelial barrier function was monitored by measuring the permeability of a large inert molecule, FD4. In line with our previously defined cut-off value of 1%/h for proper barrier integrity^[28,40]^, FD4 permeability was low, with values between 0.05%/h and 0.77%/h [Figure 3A]. Although not significantly, FD4 permeability decreased on average by 34.2% or 28.2% upon inulin treatment of the microbiome or SCFA supplementation, respectively. Permeability of two smaller molecules, antipyrine and atenolol, followed the same trend with lower 20-24 h average P_app_ values upon inulin treatment or SCFA supplementation [Figure 3B and C]. Significant results were observed for antipyrine transport by a decrease from 37.5 × 10^-6^ cm/s under control conditions to 28.3 × 10^-6^ cm/s and 28.1 × 10^-6^ cm/s upon inulin treatment or with SCFA supplementation, respectively. As FD4 and small molecule transport data for both the inulin treatment and SCFA supplementation show similar observations, these decreases are likely attributed to the different SCFA composition in these conditions compared to the control condition. By taking the different transport characteristics of antipyrine and atenolol into account, tissue functionality can be assessed^[28,40,42]^. Antipyrine is a highly permeable drug [fraction absorbed (FA) of 100%] that translocates transcellularly and atenolol is a moderately permeable drug (50% FA) that translocates paracellularly. Consequently, tissue with good functionality will show 2-fold higher P_app_ values for antipyrine than for atenolol^[28]^. Here, adequate tissue functionality was shown for all three test conditions with an antipyrine/atenolol ratio not different from 2 and no differences between the test conditions [Figure 3D]. Next, we evaluated tissue viability by measuring LDH secretion into the apical and basolateral compartments over time and determined the intracellular LDH levels at the end of the experiments. Comparable with our previous reports on LDH secretion by human or porcine colon tissue in the IEBC^[28,42]^, the cumulative LDH release was 15.6% under control conditions [Figure 3E]. Comparable levels were observed upon incubation with the inulin-treated i-screen supernatant, whereas significantly lower levels were detected upon the addition of SCFA to control i-screen supernatant. Endpoint intracellular LDH levels were highest for the inulin-treated condition, but not significantly different from control, thus indicating comparable levels of tissue viability after 24 h of incubation [Figure 3F].

### Human colon tissue secretes less pro-inflammatory cytokines when exposed to i-screen supernatant with (added) increased butyrate concentrations

**Figure 4:**
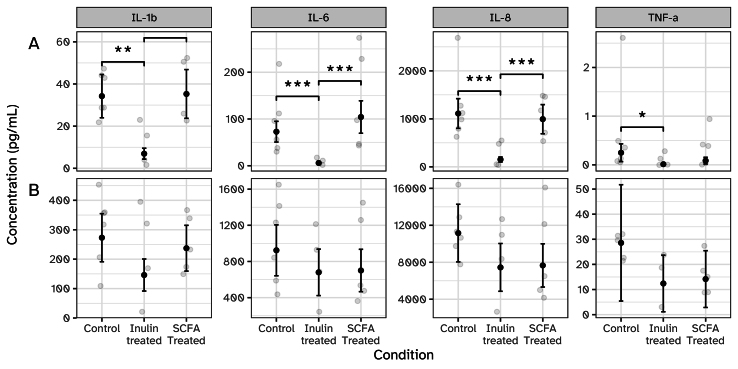
Cytokine release by human colon tissue explants (donor 3) in the IEBC exposed to i-screen supernatant (untreated control, inulin-treated, and untreated control with added SCFA) for 24 h (*n* = 5-6). (A) Secretion of IL-1β , IL-6 , IL-8 , and TNF-α into the apical compartment; (B) Secretion of IL-1β , IL-6 , IL-8 , and TNF-α into the basolateral compartment. Black error bars with points represent estimated marginal means with standard errors, obtained from linear mixed-effects models. *P < 0.05; **P < 0.01. IEBC: Intestinal explant barrier chip; SCFA: short-chain fatty acid.

SCFAs, particularly butyrate, are known for having anti-inflammatory effects in the gut^[45,46]^. Thus, we evaluated the secretion of a broad panel of pro- and anti-inflammatory cytokines by the human colon tissue explants, as their levels can be indicative of changes in the inflammatory state of the tissue. Communication of the epithelial cells to immune cells occurs predominantly at the basolateral side of the tissue. Correspondingly, all cytokines were detected at higher concentrations in the basolateral medium than in the apical medium [Figure 4A and B, Supplementary Figure 1]. The cytokines for which the highest concentrations were detected were IL-1β, IL-6, IL-8, and TNF-α. For the other six cytokines, IFN-γ, IL-10, IL-12p70, IL-13, IL-2, and IL-4 values were low (< 10 pg/mL) and therefore they might be considered to be less relevant. At the basolateral side, both inulin and SCFA treatments show a trend to decrease the concentration of IL-1β, IL-6, IL-8, and TNF-α. At the apical side, the concentrations of the same four cytokines were significantly decreased for the inulin-treated condition, but not for the SCFA treatment. These observations might indicate that although the SCFAs that are present in both conditions have a potentially beneficial effect on the release of pro-inflammatory cytokines at the basolateral side, another component is likely responsible for the apical decrease of the release of these cytokines in the inulin-treated condition. Additionally, the concentration of 5 of the other 6 cytokines tested, namely IFN-γ, IL-10, IL-12p70, IL-2, and IL-4, were also lower upon exposure to the inulin-treated i-screen supernatant or SCFA-supplemented i-screen control supernatant compared to the control condition.

### mRNA gene expression profiles confirm increased barrier function and anti-inflammatory effect upon inulin treatment or SCFA supplementation

**Figure 5:**
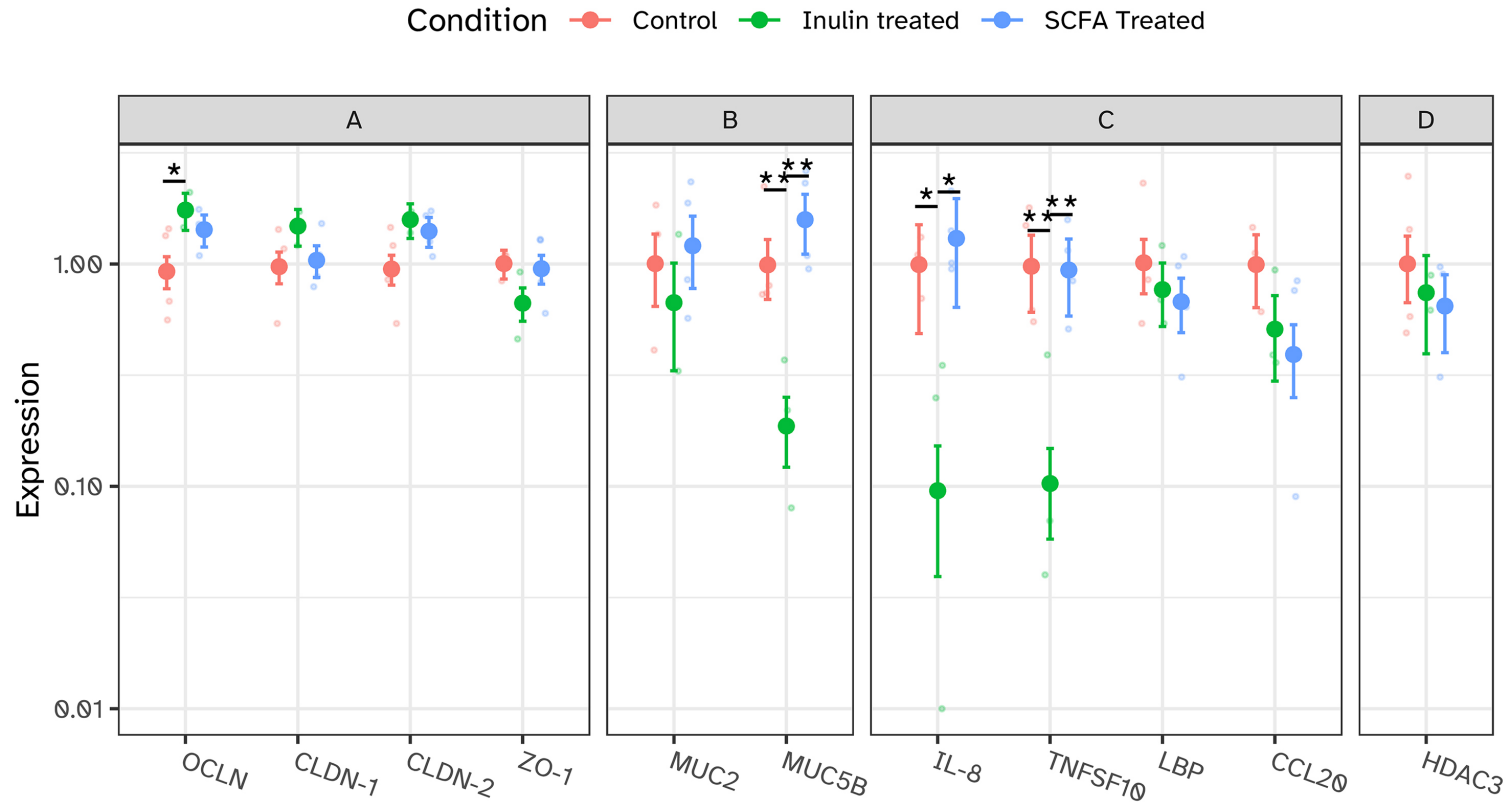
mRNA expression levels of 11 genes determined by RT-qPCR in human colon tissue explants (donor 3) in the IEBC exposed to i-screen supernatant (untreated control, inulin-treated, and untreated control with added SCFA) for 24 h (*n* = 5-6). (A) Tight junction complex genes OCLN, CLDN-1, CLDN-2, and ZO-1; (B) Mucus genes mucin MUC2 and mucin MUC5B; (C) Genes related to the inflammatory response: cytokine IL-8, TNFSF10, LBP, and CCL20; (D) Chromatin remodeler HDAC3. Target gene expression is expressed relative to the reference gene 36B4 and normalized to control. Error bars with points represent estimated marginal means with standard errors, obtained from linear mixed-effects models. *P < 0.05; **P < 0.01. RT-qPCR: Reverse transcription quantitative PCR; IEBC: intestinal explant barrier chip; SCFA: short-chain fatty acid; OCLN: occludin; CLDN-1: claudin-1; CLDN-2: claudin-2; ZO-1: zonulin-1; IL-8: interleukin-8; TNFSF10: TNF superfamily member 10; LBP: LPS binding protein; CCL20: C-C motif chemokine ligand 20; HDAC3: histone deacetylase 3.

To establish whether the protective effects of the microbial inulin treatment or SCFA supplementation on tissue barrier integrity and inflammation were caused by genetic changes, we assessed the mRNA expression profiles of several genes involved in these processes. The transmembrane proteins Occludin, Claudin-1, and Claudin-2, and the cytoplasmic scaffolding protein ZO-1 are the main components of the tight junction complex of intestinal epithelial cells^[47]^. Exposure of human colon tissue segments to the inulin-treated i-screen supernatant significantly increased the mRNA expression of the major stabilizing factor of the tight junction complex occludin^[48]^ [Figure 5A], as did SCFA supplementation in a non-significant way. Claudin-1 and Claudin-2 expression were also both non-significantly higher in the segments exposed to the inulin-treated supernatant, whereas zonulin-1 showed a decreasing trend [Figure 5A]. Although a decrease of the latter sounds counterintuitive in order to protect the barrier integrity, high expression levels of zonulin-1 correlate with increased intestinal permeability as demonstrated in patients suffering from intestinal permeability disorders such as celiac disease^[49,50]^. SCFA supplementation also showed a trend to increase claudin-2 expression, but did not alter mRNA levels of claudin-1 or zonulin-1, indicating that other components than SCFA in the inulin-treated i-screen supernatant are likely at play in regulating the expression of these two genes. In addition to tight junction proteins that can be considered parts of the mechanical barrier, intestinal mucus, including mucins, adds to the chemical barrier of the gut^[51]^. Gene expression levels of the two predominantly secreted mucins in the colon, MUC2 and MUB5B^[52]^, indicated opposite effects for the inulin-treated i-screen supernatant condition compared to SCFA supplementation [Figure 5B]. MUC5B significantly decreased and MUC2 showed a trend to decrease upon exposure to the inulin-treated i-screen supernatant, whereas both genes showed a trend to increase upon exposure to control supernatant supplemented with SCFA. Mucin changes do not only alter the physical intestinal barrier, but will also impact the innate immune response^[53]^. To further map genetic changes related to inflammation, we first assessed the mRNA gene expression level of IL-8, the cytokine with the highest concentrations measured in the apical and basolateral supernatants [Figure 4]. In line with the cytokine release, mRNA expression of IL-8 was significantly reduced in the condition exposed to the inulin-treated i-screen supernatant [Figure 5C]. Inulin-treated i-screen supernatant also significantly reduced TNFSF10 (TRAIL) mRNA gene expression, another key mediator of the innate immune response^[54,55]^. IL-8 predominantly targets neutrophil attraction in the innate immune response^[56]^, whereas TNFSF10 (TRAIL) is involved in regulating controlled cell death by apoptosis and its downregulation is associated with reduced inflammation^[54,55]^. Neither IL-8 nor TNFSF10 were affected by SCFA supplementation. In contrast, the mRNA expression of LBP, an acute-phase protein of the innate immune response^[57]^, and CCL20, a factor involved in the adaptive immune response by recruiting dendritic and Th2 cells^[58,59]^, was lower, but not significantly different, for both the inulin-treated i-screen supernatant and SCFA supplementation conditions [Figure 5B], hinting towards shared mechanisms between these conditions to reduce inflammation in the gut. As an altered SCFA profile with increased amounts of butyrate is shared between the inulin-treated i-screen supernatant condition and SCFA-supplemented condition, we assessed the mRNA gene expression levels of a commonly known gene targeted by butyrate, *HDAC3*^[60,61]^. In line with the expectation that increased levels of butyrate inhibit *HDAC3*, both inulin-treated i-screen supernatant and SCFA-supplemented conditions showed a trend to decrease *HDAC3* gene expression levels [Figure 5D], thereby confirming a common initiating factor being present in these two conditions.

## DISCUSSION

In this study, we demonstrated the implementation of our i-screen^[29]^ and IEBC technologies^[28]^ in sequence as a novel efficient approach to study the interaction of microbial metabolites with the host gut tissue *ex vivo*. Over recent years, there has been an increasing incidence and emerging healthcare costs to treat and alleviate symptoms of patients with common diseases of modern society that have been associated with a dysregulation of the host-bacteria homeostasis, like obesity, metabolic syndrome, diabetes, allergies, autoimmune disorders, colorectal cancer, and IBD^[62-65]^. This means that there is a big window of opportunity to investigate novel treatment strategies based on the modulation of host-microbe interactions in the gut. Although modulation of the gut microbiome to improve health and even prevent or reduce disease, such as through pre-/pro-/antibiotics or fecal microbiota transplantation (FMT), is not new, evidence on the exact mechanism at the local interaction site is still scarce^[66]^. *In vitro* or *ex vivo* co-culture models to study the mode of action of intestinal host-microbe interactions are hardly established, hampered by the inevitable need for an anaerobic environment to culture the full gut microbiome in all its complexity, and the rapid overgrowth of intestinal cells by bacteria in static culture systems^[67]^. Using cell-free culture supernatant, also called bacteria-free supernatant, fecal supernatant, or simply supernatant, has proven to be an effective way to study host-microbe, or in fact host-microbial metabolite, interactions. For example, a few studies report on the protective effect of probiotic culture supernatants against invasion of pathogenic *Escherichia coli* (*E. coli*) strains or against 5-fluorouracil (5-FU)-induced intestinal epithelial cell damage^[68-70]^. The latter study used an intestinal epithelial cell line derived from rat intestine^[68]^, wherefore results might be less relevant for humans, while the models that applied an *E. coli* challenge used either the human intestine-derived HT-29 cell line^[70]^ or Caco-2 and T84 cell lines^[69]^. One step further towards the physiological resemblance of the human intestinal tissue structure goes a Caco-2/dendritic cell co-culture model that studies the innate immune response towards a pathogenic challenge with *Salmonella typhi*^[71]^. Still, by using cell lines and supernatants of single probiotic strains, these *in vitro* models have a rather limited representation of the complex situation *in vivo* with respect to both the gut tissue and the microbiome. Both aspects were grasped in their full complexity in a recent study by Gonzales *et al.*, where they showed impaired intestinal digestive and barrier function upon transferring fecal supernatant of human Autism Spectrum Disorder patients into mice^[72]^. Although mouse models are frequently employed in biomedical research and allow for studying host-microbe interactions in a controlled setting, using a mouse model for human gut microbiota research has often limited translatability^[73]^. Therefore, we developed a co-culture model in which both the human microbiome and the human gut tissue architecture were fully represented. In fact, to the best of our knowledge, this is the first gut-on-a-chip model with fresh tissue explants that studied intestinal host-microbial metabolite interactions. Furthermore, this is the only gut-on-a-chip model where the intestinal compartment is directly exposed to the supernatant of the complete microbiome, without, for example, a membrane for separation of the microbial and intestinal chambers^[74,75]^. Although we also have a static model with fresh intestinal tissue explants, the InTESTine^[40,43]^, we chose to use our IEBC gut-on-a-chip model as the microfluidic flow enhances tissue viability and mucus formation^[28,67]^. These advantages and the two-compartmentalized set-up have popularized the gut-on-a-chip technology for intestinal host-microbe interaction studies over the recent years, yielding a range of different designs. The establishment of an aerobic-anaerobic interface seems to be the most sought-after design and would be even more physiologically relevant than using culture supernatant, as such an interface would enable the co-culture of the anaerobic microbiome with aerobic gut cells or tissue. However, the variety of technologies and low number of publications show that this is a complicated research area^[67,76]^. The model presented here might bridge the gap until such an aerobic-anaerobic interface platform is successfully developed and can fulfill the need to study host-microbe, or host-microbial metabolites, interactions in the adult human intestine. Still, we realize that even the current set-up might need adaptions to further improve its physiological resemblance, e.g., by decreasing the apical pH from 6.5 to 6.0, lowering the glucose concentration in the media, and removing or studying the effect of the antimicrobials in the i-screen supernatant on the existing microbiome of the tissue explants. Nevertheless, by varying the source of the microbiome, e.g., from diseased populations, or fresh tissue explants, e.g., from a young patient, this model can be adapted to fit a variety of research questions.

The major findings of the data presented here are that the prebiotic fiber inulin shifted the microbiome composition towards a more butyrogenic composition after 24 h of incubation, and that the consequently elevated butyrate levels likely played an active role in the increase of the intestinal barrier function and reduced inflammation in human colon tissue explants. Bacterial fermentation of the prebiotic fiber inulin leads to the generation of SCFAs^[13]^ and can promote the growth of, e.g., *Bifidobacterium* and butyrate-producing species such as *Faecalibacterium*, *Roseburia*, and *Anaerostipes*^[19,24,77,78]^. Here, we used a concentration of 4 mg/mL because the expected concentration of prebiotic products (oral dose 1-10 g) in the colon is likely between 1-10 mg/mL and previous *in vitro* studies have found clear prebiotic effects with this concentration^[30]^. We found that supplementation of culture media with inulin shifted the gut microbial community towards a higher relative abundance of *Bifidobacterium*, *Anaerostipes*, *Blautia*, and *Collinsella*. These bacterial groups are generally more abundant in the microbiomes of healthy people than in those of diseased people and are known for their capacity to produce SCFAs^[79]^. Furthermore, the addition of inulin to the microbiome culture medium increased the total level of SCFAs and generated an increase in the contribution of butyrate to the total SCFA pool, as we have seen before^[20]^. In fact, the relative contribution of butyrate more than doubled from 10% to 22%. The contribution of propionate decreased from 20% to 12%, and the acetate ratio remained more or less the same (63% *vs.* 64%) and in line with the average level of acetate in the human gut^[14,17]^. Although most gut microbiota species can produce acetate, the production pathways for propionate and butyrate are more conserved^[14,17]^. Propionate is produced via the succinate pathway, used by *Bacteroidota* and many *Negativicutes*, or via the acrylate and propanediol pathways restricted to the *Lachnospiraceae* and *Negativicutes*^[14,17]^. Butyrate production occurs via butyrate kinase or butyryl-CoA:acetate-CoA transferase, the latter being the major pathway and needing the presence of acetate^[14]^. The main butyrate producers belong to *Faecalibacterium*, *Eubacterium*, *Roseburia*, *Coprococcus*, *Anaerostipes*, *Subdoligranulum*, and *Anaerobutyricum* genera^[14,17,80]^. Indeed, significant changes in microbiome composition caused by inulin supplementation in the i-screen affected most of these genera, with *Anaerostipes* and *Coprocococcus* showing the highest increase in relative abundance. Along with an increase in butyrogenic bacteria, the relative abundance of putative propionate-producing bacteria such as *Bacteroidota* was reduced after 24 h, reflecting our findings in the SCFA composition shift. In addition, the relative abundance of *Bifidobacterium* increased significantly, whereas *Escherichia* and *Shigella* decreased. Although most species of the latter two genera are harmless, *Escherichia* or *Shigella* intestinal overgrowth is associated with diarrhoeal disease and thus their reduction can be seen as beneficial^[81,82]^. Bifidobacteria are generally viewed as having health benefits^[83,84]^. An increase in bifidobacteria is often associated with an increase in butyrate, not through butyric acid production by bifidobacteria themselves but rather in association with cross-feeding mechanisms in co-culture with other bacteria^[85]^. This will be an interesting mechanism to study in future studies. Our observations are in line with human clinical trial data for inulin, which show a comparable change in microbiome composition towards increased *Bifidobacterium*, *Anaerostipes*, and *Faecalibacterium*, and decreased *Bacteroides*, but could not always confirm that these changes were associated with increased SCFA levels^[21-24]^.

*In vivo*, SCFAs are readily absorbed by the host. In total, 90%-95% of SCFA are absorbed by the gut epithelial cells, enter the systemic circulation, and exert their effects via different signaling pathways, or, in the case of butyrate, can also be used as an energy source for colonocytes^[15,16]^. Studying the effects of microbial metabolites such as SCFAs can be facilitated using *in vitro* or *ex vivo* model technologies such as the IEBC gut-on-a-chip model presented in this study. The lower permeability of the human colon tissue for both the large molecule FD4 and the small molecules antipyrine and atenolol hint towards a tighter epithelial barrier. This effect was observed after both the inulin treatment and SCFA supplementation, and thus is likely attributed to the shift in SCFA composition compared to the control situation. Gene expression data of major tight junction proteins confirmed the presence of a tighter epithelial barrier with an upregulation of OCLN and CLDN-2, but also highlighted that the i-screen supernatant induced additional beneficial effects for CLDN-1 and ZO-1 compared to SCFA supplementation alone. With increased butyrate levels as a common denominator, the shared effects of the two exposure conditions are likely attributed to it. Indeed, butyrate is well known for its stimulating effects on intestinal epithelial barrier function and immune function and, therefore, can be considered a therapeutic intervention for IBD or other gastrointestinal diseases with a hampered barrier or immune activity^[47,86-89]^. Several *in vitro* studies using Caco-2, HT-29, or other human-derived cell lines support the barrier-strengthening effect of butyrate^[90-92]^, but so far, this has not yet been confirmed in *ex vivo* human tissue. The only other study to date identified by these authors employing fresh human gut tissue explants to study the effect of butyrate on intestinal barrier function, could not see any difference of a 5 or 25 mM butyrate treatment on paracellular or transcellular permeability, nor at the gene expression level of tight junction proteins^[93]^. The higher concentration of 25 mM is comparable to the butyrate concentration in the inulin-treated and SCFA-supplemented conditions in this study. Of note, the incubation time in that study was only 1 h, a known limitation of the Ussing technology^[94]^, and might have been too short for butyrate to exert its effects. Additionally, butyrate was applied together with a stressor that caused a leaky gut, Compound 48/80^[93]^, which is an important difference from the set-up of this study in which the tissue explants were not challenged by a hyperpermeability inducer. So even though we could already demonstrate the ameliorating effect of the butyrate-dominated SCFA-composition shift on intestinal barrier function, demonstrating a preventative or treatment effect of the enriched i-screen supernatants when applying a challenge to the intestinal tissue explants in the IEBC system would be a very interesting topic for future experiments. Butyrate is also well-known for its anti-inflammatory effects^[45,46,87,88,95]^. Butyrate can modulate the innate and adaptive immune system by influencing neutrophils, macrophages, and T-cells^[17]^ and via downregulation of histone deacetylase (HDAC)^[96-99]^, leading to lower TNF-α levels^[98]^, higher IL-10 levels^[99,100]^, and increased numbers of regulatory T-cells^[101]^. Here, we measured cytokine levels in the tissue supernatant as cytokine levels can indicate changes in the inflammation state of the tissue explants. Furthermore, in the tissue itself, we measured mRNA gene expression levels of several genes related to inflammation. We showed reduced release of TNF-α, and of other pro-inflammatory cytokines, at the basolateral (systemic) side of the intestinal tissue explants as well as downregulated *HDAC3* gene expression upon exposure to the inulin-treated and SCFA-supplemented supernatants, thereby likely confirming the anti-inflammatory role of butyrate. However, for the condition exposed to the inulin-treated i-screen supernatant, additional and significant anti-inflammatory effects on the apical (luminal) pro-inflammatory cytokine release and mRNA gene expression were observed. The expression of MUC genes was also only affected by this condition. These observations imply that a factor other than butyrate is at play, uniquely present in the inulin-treated i-screen supernatant. Of all metabolites produced by the microbiome, we only measured the SCFA and BCFA concentrations in this study. As BCFA concentrations could not be matched in the SCFA-supplemented condition (as they were lower in the inulin-treated condition and could not be removed from the supernatant), the significantly lower concentrations of iso-butyrate and iso-valerate concentrations in the inulin-treated condition could have contributed to its observed beneficial effects. Indeed, it is described that IBD patients have a 25% higher production of these BCFA^[102]^ and *in vitro* experiments with high concentrations of iso-valerate stimulated the release of pro-inflammatory cytokines^[103]^. However, not many other (*in vitro*) studies have been performed with these BCFA and additionally iso-butyrate is poorly metabolized by enterocytes^[104,105]^; thus, the potential effect of BCFA remains rather speculative here. Likely, another metabolite, or group of metabolites, contributes to the additional positive effects of the inulin-treated supernatant on intestinal barrier function and inflammation. In the future, therefore, we might apply untargeted metabolomics to identify the different metabolites produced by the microbiome in response to inulin. Studying the metabolome, the collection of metabolites with usually a low molecular weight (< 2,000 Da), has become a popular analytical approach to identify and quantify novel biomarkers for human health and disease, to detect responses to drug interventions or (environmental) stressors, and to characterize microbial metabolism^[106,107]^. It is a sport in itself to perform metabolomic research using complicated techniques such as nuclear magnetic resonance (NMR) spectroscopy or mass spectroscopy (MS), often coupled to liquid chromatography (LC) or other chromatography techniques for separation^[108,109]^. Therefore, doing this body work goes beyond the scope of the current study, but would be one of the first steps to take in the future.

In conclusion, our findings are in line with *in vitro*, *ex vivo*, and *in vivo* literature and show that the combination of i-screen and IEBC technologies provides a novel and effective way to study complex intestinal host-microbe interactions and the impact of these interactions on gut health and host wellness.

## DECLARATIONS

### Availability of data and materials

Raw data files are available upon request.

### Copyright

© The Author(s) 2024.

## References

[1] Geng ZH, Zhu Y, Li QL, Zhao C, Zhou PH (2022). Enteric nervous system: the bridge between the gut microbiota and neurological disorders. Front Aging Neurosci.

[2] Tokuhara D, Kurashima Y, Kamioka M, Nakayama T, Ernst P, Kiyono H (2019). A comprehensive understanding of the gut mucosal immune system in allergic inflammation. Allergol Int.

[3] Fung TC, Olson CA, Hsiao EY (2017). Interactions between the microbiota, immune and nervous systems in health and disease. Nat Neurosci.

[4] Sekirov I, Russell SL, Antunes LCM, Finlay BB (2010). Gut microbiota in health and disease. Physiol Rev.

[5] O’Hara AM, Shanahan F (2006). The gut flora as a forgotten organ. EMBO Rep.

[6] Erny D, Hrabě de Angelis AL, Prinz M (2017). Communicating systems in the body: how microbiota and microglia cooperate. Immunology.

[7] Hou K, Wu ZX, Chen XY (2022). Microbiota in health and diseases. Signal Transduct Target Ther.

[8] Gomaa EZ (2020). Human gut microbiota/microbiome in health and diseases: a review. Antonie Van Leeuwenhoek.

[9] Hooper LV, Littman DR, Macpherson AJ (2012). Interactions between the microbiota and the immune system. Science.

[10] Sommer F, Bäckhed F (2013). The gut microbiota - masters of host development and physiology. Nat Rev Microbiol.

[11] Clemente JC, Ursell LK, Parfrey LW, Knight R (2012). The impact of the gut microbiota on human health: an integrative view. Cell.

[12] Rinninella E, Raoul P, Cintoni M (2019). What is the healthy gut microbiota composition? A changing ecosystem across age, environment, diet, and diseases. Microorganisms.

[13] Roberfroid M, Slavin J (2000). Nondigestible oligosaccharides. Crit Rev Food Sci Nutr.

[14] Blaak EE, Canfora EE, Theis S (2020). Short chain fatty acids in human gut and metabolic health. Benef Microbes.

[15] Parada Venegas D, De la Fuente MK, Landskron G (2019). Corrigendum: short chain fatty acids (SCFAs)-mediated gut epithelial and immune regulation and its relevance for inflammatory bowel diseases. Front Immunol.

[16] Yu X, Gurry T, Nguyen LTT, Richardson HS, Alm EJ (2020). Prebiotics and community composition influence gas production of the human gut microbiota. mBio.

[17] Deleu S, Machiels K, Raes J, Verbeke K, Vermeire S (2021). Short chain fatty acids and its producing organisms: an overlooked therapy for IBD?. EBioMedicine.

[18] Chen T, Long W, Zhang C, Liu S, Zhao L, Hamaker BR (2017). Fiber-utilizing capacity varies in *Prevotella*- versus *Bacteroides*-dominated gut microbiota. Sci Rep.

[19] Gibson GR, Hutkins R, Sanders ME (2017). Expert consensus document: The International Scientific Association for Probiotics and Prebiotics (ISAPP) consensus statement on the definition and scope of prebiotics. Nat Rev Gastroenterol Hepatol.

[20] Fehlbaum S, Prudence K, Kieboom J (2018). *In vitro* fermentation of selected prebiotics and their effects on the composition and activity of the adult gut microbiota. Int J Mol Sci.

[21] Vandeputte D, Falony G, Vieira-Silva S (2017). Prebiotic inulin-type fructans induce specific changes in the human gut microbiota. Gut.

[22] Baxter NT, Schmidt AW, Venkataraman A, Kim KS, Waldron C, Schmidt TM (2019). Dynamics of human gut microbiota and short-chain fatty acids in response to dietary interventions with three fermentable fibers. mBio.

[23] Wang X, Wang T, Zhang Q, Xu L, Xiao X (2021). Dietary supplementation with inulin modulates the gut microbiota and improves insulin sensitivity in prediabetes. Int J Endocrinol.

[24] (2020). Le Bastard Q, Chapelet G, Javaudin F, Lepelletier D, Batard E, Montassier E. The effects of inulin on gut microbial composition: a systematic review of evidence from human studies. Eur J Clin Microbiol Infect Dis.

[25] Holscher HD (2017). Dietary fiber and prebiotics and the gastrointestinal microbiota. Gut Microbes.

[26] McDonald JAK (2017). *In vitro* models of the human microbiota and microbiome. Emerg Top Life Sci.

[27] Rahman S, Ghiboub M, Donkers JM (2021). The progress of intestinal epithelial models from cell lines to gut-on-chip. Int J Mol Sci.

[28] Eslami Amirabadi H, Donkers JM, Wierenga E (2022). Intestinal explant barrier chip: long-term intestinal absorption screening in a novel microphysiological system using tissue explants. Lab Chip.

[29] Schuren F, Agamennone V, Keijser B, Abeln E, van der Vossen J, Montijn R (2019). The i-screen: a versatile preclinical platform for gut microbiota studies. J Prob Health.

[30] Ladirat SE, Schols HA, Nauta A (2013). High-throughput analysis of the impact of antibiotics on the human intestinal microbiota composition. J Microbiol Methods.

[31] Wiese M, Schuren FHJ, Smits WK (2022). 2’-Fucosyllactose inhibits proliferation of *Clostridioides difficile* ATCC 43599 in the CDi-screen, an *in vitro* model simulating *Clostridioides difficile* infection. Front Cell Infect Microbiol.

[32] Agamennone V, van den Broek TJ, de Kat Angelino-Bart A, Hoevenaars FPM, van der Kamp JW, Schuren FHJ (2023). Individual and group-based effects of in vitro fiber interventions on the fecal microbiota. Microorganisms.

[33] https://www.R-project.org/.

[34] Wickham H

[35] McMurdie PJ, Holmes S (2013). phyloseq: an R package for reproducible interactive analysis and graphics of microbiome census data. PLoS One.

[36] Stacklies W, Redestig H, Scholz M, Walther D, Selbig J (2007). pcaMethods - a bioconductor package providing PCA methods for incomplete data. Bioinformatics.

[37] Oksanen J, Simpson GL, Blanchet FG https://cran.r-project.org/web/packages/vegan/vegan.pdf.

[38] Robinson MD, McCarthy DJ, Smyth GK (2010). edgeR: a Bioconductor package for differential expression analysis of digital gene expression data. Bioinformatics.

[39] Hoffman GE, Roussos P (2021). Dream: powerful differential expression analysis for repeated measures designs. Bioinformatics.

[40] Stevens LJ, van Lipzig MMH, Erpelinck SLA (2019). A higher throughput and physiologically relevant two-compartmental human *ex vivo* intestinal tissue system for studying gastrointestinal processes. Eur J Pharm Sci.

[41] Hatton GB, Yadav V, Basit AW, Merchant HA (2015). Animal farm: considerations in animal gastrointestinal physiology and relevance to drug delivery in humans. J Pharm Sci.

[42] Donkers JM, Höppener EM, Grigoriev I (2022). Advanced epithelial lung and gut barrier models demonstrate passage of microplastic particles. Micropl Nanopl.

[43] Westerhout J, van de Steeg E, Grossouw D (2014). A new approach to predict human intestinal absorption using porcine intestinal tissue and biorelevant matrices. Eur J Pharm Sci.

[44] Hoffman GE, Schadt EE (2016). variancePartition: interpreting drivers of variation in complex gene expression studies. BMC Bioinformatics.

[45] Vinolo MAR, Rodrigues HG, Nachbar RT, Curi R (2011). Regulation of inflammation by short chain fatty acids. Nutrients.

[46] Yao Y, Cai X, Fei W, Ye Y, Zhao M, Zheng C (2022). The role of short-chain fatty acids in immunity, inflammation and metabolism. Crit Rev Food Sci Nutr.

[47] Chelakkot C, Ghim J, Ryu SH (2018). Mechanisms regulating intestinal barrier integrity and its pathological implications. Exp Mol Med.

[48] Cummins PM (2012). Occludin: one protein, many forms. Mol Cell Biol.

[49] Fasano A, Not T, Wang W (2000). Zonulin, a newly discovered modulator of intestinal permeability, and its expression in coeliac disease. Lancet.

[50] (2020). Wood Heickman LK, DeBoer MD, Fasano A. Zonulin as a potential putative biomarker of risk for shared type 1 diabetes and celiac disease autoimmunity. Diabetes Metab Res Rev.

[51] Schierack P, Nordhoff M, Pollmann M (2006). Characterization of a porcine intestinal epithelial cell line for in vitro studies of microbial pathogenesis in swine. Histochem Cell Biol.

[52] Walsh MD, Clendenning M, Williamson E (2013). Expression of MUC2, MUC5AC, MUC5B, and MUC6 mucins in colorectal cancers and their association with the CpG island methylator phenotype. Mod Pathol.

[53] Moncada DM, Kammanadiminti SJ, Chadee K (2003). Mucin and Toll-like receptors in host defense against intestinal parasites. Trends Parasitol.

[54] Sag D, Ayyildiz ZO, Gunalp S, Wingender G (2019). The role of TRAIL/DRs in the modulation of immune cells and responses. Cancers.

[55] Falschlehner C, Schaefer U, Walczak H (2009). Following TRAIL’s path in the immune system. Immunology.

[56] Bernhard S, Hug S, Stratmann AEP (2021). Interleukin 8 elicits rapid physiological changes in neutrophils that are altered by inflammatory conditions. J Innate Immun.

[57] Meng L, Song Z, Liu A, Dahmen U, Yang X, Fang H (2021). Effects of lipopolysaccharide-binding protein (LBP) single nucleotide polymorphism (SNP) in infections, inflammatory diseases, metabolic disorders and cancers. Front Immunol.

[58] Weckmann M, Collison A, Simpson JL (2007). Critical link between TRAIL and CCL20 for the activation of T_H_2 cells and the expression of allergic airway disease. Nat Med.

[59] Sierro F, Dubois B, Coste A, Kaiserlian D, Kraehenbuhl JP, Sirard JC (2001). Flagellin stimulation of intestinal epithelial cells triggers CCL20-mediated migration of dendritic cells. Proc Natl Acad Sci U S A.

[60] Schilderink R, Verseijden C, Seppen J (2016). The SCFA butyrate stimulates the epithelial production of retinoic acid via inhibition of epithelial HDAC. Am J Physiol Gastrointest Liver Physiol.

[61] Chriett S, Dąbek A, Wojtala M, Vidal H, Balcerczyk A, Pirola L (2019). Prominent action of butyrate over β-hydroxybutyrate as histone deacetylase inhibitor, transcriptional modulator and anti-inflammatory molecule. Sci Rep.

[62] Hecker J, Freijer K, Hiligsmann M, Evers SMAA (2022). Burden of disease study of overweight and obesity; the societal impact in terms of cost-of-illness and health-related quality of life. BMC Public Health.

[63] (2022). van den Broek-Altenburg E, Atherly A, Holladay E. Changes in healthcare spending attributable to obesity and overweight: payer- and service-specific estimates. BMC Public Health.

[64] Singh S, Qian AS, Nguyen NH (2022). Trends in U.S. health care spending on inflammatory bowel diseases, 1996-2016. Inflamm Bowel Dis.

[65] https://www.cdc.gov/chronicdisease/about/costs/index.htm.

[66] Quaranta G, Guarnaccia A, Fancello G (2022). Fecal microbiota transplantation and other gut microbiota manipulation strategies. Microorganisms.

[67] Donkers JM, van der Vaart JI, van de Steeg E (2023). Gut-on-a-chip research for drug development: implications of chip design on preclinical oral bioavailability or intestinal disease studies. Biomimetics.

[68] Wang H, Bastian SEP, Cheah KY, Lawrence A, Howarth GS (2014). *Escherichia coli* Nissle 1917-derived factors reduce cell death and late apoptosis and increase transepithelial electrical resistance in a model of 5-fluorouracil-induced intestinal epithelial cell damage. Cancer Biol Ther.

[69] Khodaii Z, Ghaderian SMH, Natanzi MM (2017). Probiotic bacteria and their supernatants protect enterocyte cell lines from enteroinvasive Escherichia coli (EIEC) invasion. Int J Mol Cell Med.

[70] Rocha-Ramírez LM, Hernández-Chiñas U, Moreno-Guerrero SS, Ramírez-Pacheco A, Eslava CA (2023). In vitro effect of the cell-free supernatant of the *Lactobacillus casei* strain IMAU60214 against the different pathogenic properties of Diarrheagenic *Escherichia coli*. Microorganisms.

[71] Bermudez-Brito M, Muñoz-Quezada S, Gómez-Llorente C, Matencio E, Romero F, Gil A (2015). *Lactobacillus paracasei* CNCM I-4034 and its culture supernatant modulate *Salmonella*-induced inflammation in a novel transwell co-culture of human intestinal-like dendritic and Caco-2 cells. BMC Microbiol.

[72] Gonzales J, Marchix J, Aymeric L (2021). Fecal supernatant from adult with autism spectrum disorder alters digestive functions, intestinal epithelial barrier, and enteric nervous system. Microorganisms.

[73] Nguyen TLA, Vieira-Silva S, Liston A, Raes J (2015). How informative is the mouse for human gut microbiota research?. Dis Model Mech.

[74] Marzorati M, Vanhoecke B, De Ryck T (2014). The HMI™ module: a new tool to study the Host-Microbiota Interaction in the human gastrointestinal tract *in vitro*. BMC Microbiol.

[75] Shah P, Fritz JV, Glaab E (2016). A microfluidics-based *in vitro* model of the gastrointestinal human-microbe interface. Nat Commun.

[76] Morelli M, Kurek D, Ng CP, Queiroz K (2023). Gut-on-a-chip models: current and future perspectives for host - microbial interactions research. Biomedicines.

[77] Scott KP, Martin JC, Duncan SH, Flint HJ (2014). Prebiotic stimulation of human colonic butyrate-producing bacteria and bifidobacteria, *in vitro*. FEMS Microbiol Ecol.

[78] Sawicki CM, Livingston KA, Obin M, Roberts SB, Chung M, McKeown NM (2017). Dietary fiber and the human gut microbiota: application of evidence mapping methodology. Nutrients.

[79] Scotti E, Boué S, Sasso GL (2017). Exploring the microbiome in health and disease: implications for toxicology. Toxicol Res Appl.

[80] Singh V, Lee G, Son H (2022). Butyrate producers, “The Sentinel of Gut”: their intestinal significance with and beyond butyrate, and prospective use as microbial therapeutics. Front Microbiol.

[81] Belotserkovsky I, Sansonetti PJ, Jenkins C

[82] dos Reis RS, Horn F (2010). Enteropathogenic *Escherichia coli*, *Samonella*, *Shigella* and *Yersinia*: cellular aspects of host-bacteria interactions in enteric diseases. Gut Pathog.

[83] Chen J, Chen X, Ho CL (2021). Recent development of probiotic *Bifidobacteria* for treating human diseases. Front Bioeng Biotechnol.

[84] O’Callaghan A, van Sinderen D (2016). Bifidobacteria and their role as members of the human gut microbiota. Front Microbiol.

[85] Belenguer A, Duncan SH, Calder AG (2006). Two routes of metabolic cross-feeding between *Bifidobacterium adolescentis* and butyrate-producing anaerobes from the human gut. Appl Environ Microbiol.

[86] Guilloteau P, Zabielski R, Hammon HM, Metges CC (2010). Nutritional programming of gastrointestinal tract development. Is the pig a good model for man?. Nutr Res Rev.

[87] Hodgkinson K, El Abbar F, Dobranowski P (2023). Butyrate’s role in human health and the current progress towards its clinical application to treat gastrointestinal disease. Clin Nutr.

[88] Anshory M, Effendi RMRA, Kalim H (2023). Butyrate properties in immune-related diseases: friend or foe?. Fermentation.

[89] Recharla N, Geesala R, Shi XZ (2023). Gut microbial metabolite butyrate and its therapeutic role in inflammatory bowel disease: a literature review. Nutrients.

[90] Peng L, He Z, Chen W, Holzman IR, Lin J (2007). Effects of butyrate on intestinal barrier function in a Caco-2 cell monolayer model of intestinal barrier. Pediatr Res.

[91] Peng L, Li ZR, Green RS, Holzman IR, Lin J (2009). Butyrate enhances the intestinal barrier by facilitating tight junction assembly via activation of AMP-activated protein kinase in Caco-2 cell monolayers. J Nutr.

[92] Kinoshita M, Suzuki Y, Saito Y (2002). Butyrate reduces colonic paracellular permeability by enhancing PPARγ activation. Biochem Biophys Res Commun.

[93] Tabat MW, Marques TM, Markgren M, Löfvendahl L, Brummer RJ, Wall R (2020). Acute effects of butyrate on induced hyperpermeability and tight junction protein expression in human colonic tissues. Biomolecules.

[94] Donkers JM, Eslami Amirabadi H, van de Steeg E (2021). Intestine-on-a-chip: next level *in vitro* research model of the human intestine. Curr Opin Toxicol.

[95] Siddiqui MT, Cresci GAM (2021). The immunomodulatory functions of butyrate. J Inflamm Res.

[96] Zheng L, Kelly CJ, Battista KD (2017). Microbial-derived butyrate promotes epithelial barrier function through IL-10 receptor-dependent repression of claudin-2. J Immunol.

[97] Ratajczak W, Rył A, Mizerski A, Walczakiewicz K, Sipak O, Laszczyńska M (2019). Immunomodulatory potential of gut microbiome-derived short-chain fatty acids (SCFAs). Acta Biochim Pol.

[98] Vinolo MAR, Rodrigues HG, Hatanaka E, Sato FT, Sampaio SC, Curi R (2011). Suppressive effect of short-chain fatty acids on production of proinflammatory mediators by neutrophils. J Nutr Biochem.

[99] Park J, Kim M, Kang SG (2015). Short-chain fatty acids induce both effector and regulatory T cells by suppression of histone deacetylases and regulation of the mTOR-S6K pathway. Mucosal Immunol.

[100] Liu L, Li L, Min J (2012). Butyrate interferes with the differentiation and function of human monocyte-derived dendritic cells. Cell Immunol.

[101] Arpaia N, Campbelle C, Fan X (2013). Metabolites produced by commensal bacteria promote peripheral regulatory T-cell generation. Nature.

[102] (2004). van Nuenen MHMC, Venema K, van Der Woude JCJ, Kuipers EJ. The metabolic activity of fecal microbiota from healthy individuals and patients with inflammatory bowel disease. Dig Dis Sci.

[103] (2005). van Nuenen MHMC, de Ligt RAF, Doornbos RP, van der Woude JCJ, Kuipers EJ, Venema K. The influence of microbial metabolites on human intestinal epithelial cells and macrophages in vitro. FEMS Immunol Med Microbiol.

[104] Dengler F, Kraetzig A, Gäbel G (2021). Butyrate protects porcine colon epithelium from hypoxia-induced damage on a functional level. Nutrients.

[105] (2022). van Deuren T, Blaak EE, Canfora EE. Butyrate to combat obesity and obesity-associated metabolic disorders: current status and future implications for therapeutic use. Obes Rev.

[106] Vernocchi P, Del Chierico F, Putignani L (2016). Gut microbiota profiling: metabolomics based approach to unravel compounds affecting human health. Front Microbiol.

[107] Bauermeister A, Mannochio-Russo H, Costa-Lotufo LV, Jarmusch AK, Dorrestein PC (2022). Mass spectrometry-based metabolomics in microbiome investigations. Nat Rev Microbiol.

[108] Segers K, Declerck S, Mangelings D, Vander Heyden Y, Van Eeckhaut A (2019). Analytical techniques for metabolomic studies: a review. Bioanalysis.

[109] Miggiels P, Wouters B, van Westen GJP, Dubbelman AC, Hankemeier T (2019). Novel technologies for metabolomics: more for less. TrAC Trend Anal Chem.

