# Supplementary material for: A host-microbial metabolite interaction gut-on-a-chip model of the adult human intestine demonstrates beneficial effects upon inulin treatment of gut microbiome
Source: Microbiome Res Rep. 2024 Feb 22;3(2):18. doi: 10.20517/mrr.2023.79 (PMC11149092; doi:10.20517/mrr.2023.79)
Supplement: Supplementary file 1 [file mrr-3-2-18-SupplementaryMaterials.pdf]

Supplementary Material

A host-microbial metabolite interaction gut-on-a-chip model of the adult human intestine demonstrates beneficial effects upon inulin treatment of gut microbiome

Joanne M. Donkers[1], Maria Wiese[2], Tim J. van den Broek[2], Esmée Wierenga[1], Valeria Agamennone[2], Frank Schuren[2], Evita van de Steeg[1]

[1]Department of Metabolic Health Research, TNO, Leiden 2333 BE, the Netherlands.
[2]Department of Microbiology & Systems Biology, TNO, Leiden 2333 BE, the Netherlands.

Correspondence to: Dr. Joanne M. Donkers, Department of Metabolic Health Research, TNO, Sylviusweg 71, Leiden 2333 BE, the Netherlands. E-mail: joanne.donkers@tno.nl

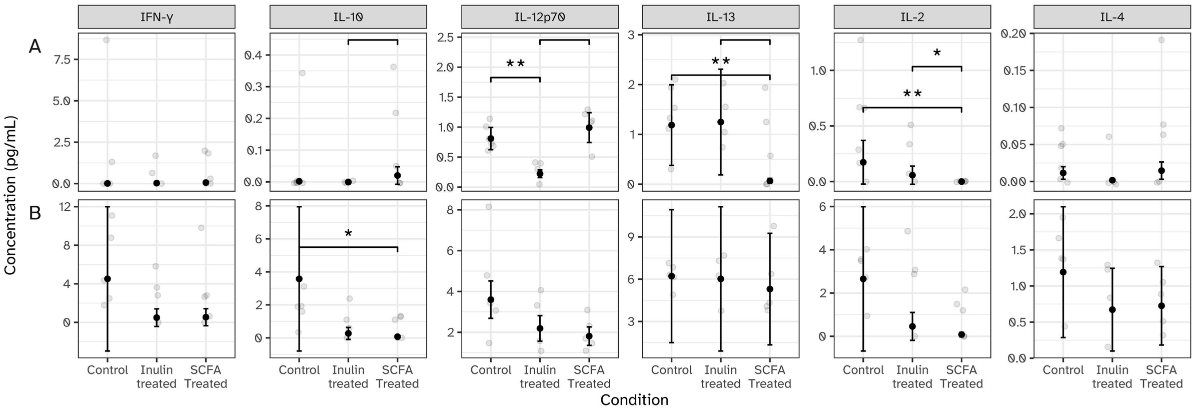

Supplementary Figure 1. Cytokine release by human colon tissue explants (donor 3) in the IEBC exposed to i-screen supernatant (untreated control, inulin-treated, and untreated control with added SCFA) for 24 h (n = 5-6). (A) Secretion of IFN-γ, IL-10, IL-12p70, IL-13, IL-2, and IL-4 into the apical compartment; (B) Secretion of IFN-γ, IL-10, IL-12p70, IL-13, IL-2, and IL-4 into the basolateral compartment. Black error bars with point represent estimated marginal means with standard errors, obtained from linear mixed-effects models. *$P < 0.05$; **$P < 0.01$.
